# Modulation of G_q_/PLC-Mediated Signaling by Acute Lithium Exposure

**DOI:** 10.3389/fncel.2022.838939

**Published:** 2022-02-15

**Authors:** Cesar Adolfo Sánchez Triviño, Maria Paula Landinez, Sara Duran, María del Pilar Gomez, Enrico Nasi

**Affiliations:** ^1^Departamento de Biología, Universidad Nacional de Colombia, Bogotá, Colombia; ^2^Centro Internacional de Física, Universidad Nacional de Colombia, Bogotá, Colombia; ^3^Marine Biological Laboratory, Woods Hole, MA, United States; ^4^Instituto de Genética, Universidad Nacional de Colombia, Bogotá, Colombia

**Keywords:** lithium, phospholipase C, Gq, calcium, SHSY5Y, HEK-293

## Abstract

Although lithium has long been one of the most widely used pharmacological agents in psychiatry, its mechanisms of action at the cellular and molecular levels remain poorly understood. One of the targets of Li^+^ is the phosphoinositide pathway, but whereas the impact of Li^+^ on inositol lipid metabolism is well documented, information on physiological effects at the cellular level is lacking. We examined in two mammalian cell lines the effect of acute Li^+^ exposure on the mobilization of internal Ca^2+^ and phospholipase C (PLC)-dependent membrane conductances. We first corroborated by Western blots and immunofluorescence in HEK293 cells the presence of key signaling elements of a muscarinic PLC pathway (M1AchR, G_q_, PLC-β1, and IP_3_Rs). Stimulation with carbachol evoked a dose-dependent mobilization of Ca, as determined with fluorescent indicators. This was due to release from internal stores and proved susceptible to the PLC antagonist U73122. Li^+^ exposure reproducibly potentiated the Ca response in a concentration-dependent manner extending to the low millimolar range. To broaden those observations to a neuronal context and probe potential Li modulation of electrical signaling, we next examined the cell line SHsy5y. We replicated the potentiating effects of Li on the mobilization of internal Ca, and, after characterizing the basic properties of the electrical response to cholinergic stimulation, we also demonstrated an equally robust upregulation of muscarinic membrane currents. Finally, by directly stimulating the signaling pathway at different links downstream of the receptor, the site of action of the observed Li effects could be narrowed down to the G protein and its interaction with PLC-β. These observations document a modulation of G_q_/PLC/IP_3_-mediated signaling by acute exposure to lithium, reflected in distinct physiological changes in cellular responses.

## Introduction

Lithium has been widely employed as a treatment of choice for bipolar disorders for well over six decades, but its exact mechanisms of action at the cellular and molecular levels have yet to be fully elucidated. The problem has been tackled primarily with biochemical approaches, and the spectrum of clues that have emerged is wide and diverse. The multiplicity of effects of Li is hardly surprising, given its ability to stabilize opposing neurological and behavioral states. For example, Li was reported to inhibit glycogen synthase kinase (GKS3; reviewed by Jope, 2003), but the detailed functional consequences could be difficult to tease out, due to the large number of direct or indirect downstream targets and regulators of GKS3 (>100; Beurel et al., 2015). One of the earliest biochemical effects of lithium to be unveiled was the depletion of inositol and inositol-monophosphate (InsP1; Allison and Stewart, 1971; Allison et al., 1976), due to the inhibition of inositol monophosphatase (Hallcher and Sherman, 1980; Inhorn and Majerus, 1987). This spawned the celebrated inositol-depletion hypothesis of Li action (Berridge et al., 1982). Notwithstanding the reproducibility of these and other biochemical effects of lithium, its effectiveness as a therapeutic agent must ultimately be understood in terms of specific alterations of the activity of neuronal cells and circuits. Amongst the processes likely to be influenced by Li, synaptic communication figures prominently; nonetheless, physiological data remain scarce. The notion that Li may impact post-synaptic responses involving the phosphoinositide signaling cascade is a logical extension of its effect on inositol metabolism (Berridge et al., 1982). Indeed, it has been shown that Li can compromise the agonist-stimulated production of inositol second-messengers, apparently not just because of depletion of the precursor (Casebolt and Jope, 1987, 1989; Godfrey et al., 1989). Li effects on phosphoinositides appear to be linked especially to cholinergic signaling because they can be blocked by muscarinic antagonists (Allison and Blisner, 1976). The issue is further complicated by reports pointing to a Li-induced *potentiation* of cholinergic activity *in vivo* (Jope, 1979; Sherman et al., 1981; Jope et al., 1986). Additional candidate targets of lithium effects include the adenylate cyclase signaling pathway (Ebstein et al., 1980; Colin et al., 1991), as well as aspects of glutamatergic neurotransmission, such as re-uptake of glutamate (Dixon and Hokin, 1998), the expression of metabotropic glutamate receptors (mGluRs; Sourial-Bassillious et al., 2009), and NMDA receptor-dependent excitotoxicity (Nonaka et al., 1998). The variety of effects and the complexity of *in vivo* assays render the task of pin-pointing specific Li mechanisms arduous, so evidence of modification of defined cellular physiological responses remains lacking. The starting point of this work was the serendipitous observation that the photocurrent of light-sensitive neurons of the lancelet—which is mediated by melanopsin tapping into the phosphoinositide pathway—is augmented upon Na replacement with Li (Peinado et al., 2015). This was not due to a greater permeation of Li *vs*. Na ions through the light-activated channels; moreover, the photo-induced mobilization of Ca from intracellular stores (Gomez and Nasi, 2009) was also up-regulated by Li^+^. This suggested that Li exerted a modulatory influence on that PLC signaling cascade. In the present work, the issue was re-examined and extended, by probing its generality in mammalian cell lines and exploring much lower Li concentrations—approaching those used for therapeutic purposes. A robust influence on PLC-dependent internal Ca release and membrane currents was documented in individual cells acutely exposed to millimolar (mM) Li; moreover, the site of action could be narrowed down to the G_q_ protein and its interaction with PLC. Establishing Li modulation of well-defined physiological processes that are important in neuronal communication can help understand its mode of action on the regulation of the activity of brain circuits.

## Materials and Methods

### Cell Culture

Two different cell lines were employed in the present study. Initial experiments were conducted on HEK 293 cells obtained from the laboratory of Dr. Marcela Camacho at the Biology Department, *Universidad Nacional de Colombia*. Subsequently, the cell line SHsy5y, derived from neuroblastoma, was utilized as a model system more pertinent to investigate Li effects in a neuronal context. This line was obtained from the laboratory of Dr. Gonzalo Arboleda, Institute of de Genetics, *Universidad Nacional de Colombia*. Both cell lines were maintained at 70–90% confluence in 25 cm^2^ culture flasks with a filter cap (Corning T25, Corning, NY) at 37°C with 5% CO_2_ in a Thermo Scientific Series 8000 incubator. The culture medium for HEK cells was High-glucose DMEM (Gibco, Thermo Fisher Scientific, Waltham, MA), while in the case of SHsy5y it was high-glucose DMEM-F12 (Gibco). Both were supplemented with L-glutamine and 10% fetal calf serum (FCS, Gibco). On a weekly basis, cultures were trypsinized (Gibco trypsin solution at 0.05%) in either HBSS-EDTA buffer or PBS (0.1 M sodium phosphate, 0.15 M NaCl, pH 7.2; Sigma-Aldrich, St. Louis, MO) at 37°C for 1–2 min, with mechanical agitation. Cells were then re-seeded at 5–10% confluence in fresh culture medium. After 8–10 passes, the cells were discarded, and a fresh aliquot from a frozen stock of cells was thawed and cultured. For physiological experiments, aliquots of freshly trypsinized cells (passes 3–9) were seeded onto 9 mm No. 1.5 coverslips, which were previously washed in methanol, rinsed in distilled water, UV-sterilized, and cultured in 24-well plates for at least two days before the measurements.

### Inmuno-Detection

#### Western Blot

Cells were cultured in T25 flasks or in plates with 9.6 cm^2^ wells (Falcon, Corning, Corning, NY). When confluence reached 70–90% the medium was replaced with cold PBS, and then with lysis solution (5 min with agitation). For soluble proteins, Pierce Gentle Lysis buffer was used (150 mM NaCl, 1 mM EDTA, pH 8.0, 5% glycerol, 25 mM Tris HCl, pH 7.4; Thermo Scientific, Waltham, MA). For integral membrane proteins, different lysis conditions were employed: non-ionic detergents NP-40 (Surfact-Amps, Thermo Scientific) or Triton X-100 (TX-100) at 1% in 150 mM NaCl, 50 mM Tris-HCl pH 7.4; alternatively, RIPA buffer was utilized (0.1% sodium deoxycholate, 0.1% SDS, 1 mM EDTA, 0.5 mM EGTA, 1% TX-100, NaCl 140 mM, 10 mM Tris-HCl, pH 8.3). All lysis buffers were supplemented with 0.2 mM phenylmethylsulfonyl fluoride (PMSF) and 1 μl/ml protein inhibitor cocktail (Sigma-Aldrich P1860). The lysate was briefly sonicated (Laboratory Supply, Inc., Hicksville, NY; 15 s), and centrifuged at 13,000 *g* for 10 min to sediment debris. The supernatant was re-suspended in 4× volume of cold acetone, incubated at −20°C for 2 h to precipitate proteins, and centrifuged at 8,000 *g* for 10 min. The pellet was air-dried before re-suspending in loading buffer (5 mM EDTA, 2% SDS, 0.01% bromophenol blue 10% glycerol, 62.5 mM Tris HCl, 1% β-mercaptoethanol, pH 6.8) and incubated briefly at 85°C before loading the wells. Proteins were separated by SDS-PAGE (6–10%) in a mini-gel apparatus (Pharmacia Biotech SE 280, Uppsala, Sweden) under constant current (20 mA), electro-transfered onto nitrocellulose membrane using either a semi-dry (SemiPhor TE 70, Amersham Biosciences, Buckinghamshire, United Kingdom; ≈0.8 mA/cm^2^, 1 h) or a wet apparatus (Mini Trans-Blot, Bio-Rad, 100 V, 1 h). The composition of the transfer buffer was: 153 mM glycine, 20 mM Tris, 20% methanol, pH 8.3. Membranes were blocked by overnight incubation with 3% bovine serum albumin (BSA) in tris-buffered saline (TBS; 50 mM Tris-Cl, 150 mM NaCl, pH 7.5) at 4°C. Subsequently, they were treated with primary antibodies in TBS (3 h at room temperature with slow agitation) washed three times, incubated with alkaline phosphatase-conjugated secondary antibodies (Promega, Madison, WI; 1:200, 1 h), washed again, and developed (Western Blue, Promega). The reaction was stopped with 5% acetic acid. Coomassie staining of additional lanes was routinely employed both to verify protein integrity, and to corroborate post-transfer the efficient migration of the proteins to the nitrocellulose membrane.

#### Immunocytochemistry

Cells cultured onto coverslips at a confluence <80% were fixed in 2% paraformaldehyde for 15 min at room temperature, washed three times in PBS, permeabilized with 0.2% Triton-X for 5 min, and blocked for 10 min in PBS with 1% goat serum and 0.002% Triton-X-100. Samples were then incubated with primary antibodies at the appropriate dilution in PBS containing 0.5% BSA and 0.002% Triton-X, either for 1 h at room temperature or overnight at 4°C. After three washes in PBS, the coverslips were incubated with secondary antibodies conjugated to Alexa Fluor 546 or 488 (Invitrogen—Molecular Probes, Eugene, OR) 1:200 for 1 h, washed again three times in PBS, and finally mounted with 50% glycerol in PBS onto microscope slides, and sealed with transparent nail polish. In negative controls, the primary antibodies were omitted. Specimens were observed in a Zeiss Axio Imager Z2 fluorescence microscope equipped with an Axiocam monochromatic camera (Zeiss, Jena, Germany). Pseudo-coloring was employed for ease of visualization. Documentation of fluorescence images was carried out under identical conditions for experimental samples and their corresponding controls; the same fields were also photographed under Nomarski optics.

#### Antibodies

The following primary antibodies were utilized:

Rabbit anti-M3-mAChR (Alomone Labs AMR-006; Jerusalem, Israel)

Rabbit anti-G_q_/α11 (Millipore 06-709; Burlington, MA)

Mouse anti-PLCβ1 (Abcam Ab21824-100; Cambridge, United Kingdom)

Goat anti-PLCβ4 (Santa Cruz sc-31765; Santa Cruz, CA)

Rabbit anti-IP_3_R type I (Novus H00003708-A01; Novus Biologicals, Centennial, CO)

Rabbit anti-IP_3_R I-II-III (Santa Cruz Biotechnology sc-28613)

Rabbit anti-PKCγ (Abcam ab71558)

### Electrophyisiological Recordings

Coverslips seeded with cells were transferred to the recording chamber mounted on the stage of an inverted microscope (either Zeiss Axiovert or Nikon Diaphot; Melville, NY). For both patch-clamp and Ca-fluorescence measurements, the chamber was continuously perfused by gravity with a standard solution containing (in mM): 140 NaCl, 5 KCl, 1.2 MgCl_2_, 2.5 CaCl_2_, 10 HEPES, 2.5 glucose, pH = 7.4. In the low-Ca solution, its concentration was reduced to 0.1 mM and that of Mg increased to 3.6 mM. A system of manifolds and reservoirs was used to change the perfusion solution. In extracellular solutions lacking calcium or sodium, these ions were replaced on an isomolar basis with Mg and Tris, respectively. Patch electrodes, fabricated from 1.5 mm borosilicate capillary glass, were filled with an “intracellular” solution containing (in mM) 132 K-gluconate, 2 MgCl_2_, 8 NaCl, 10 HEPES, 2 Na_2_-ATP, 1 EGTA, and 0.3 Na-GTP. Electrode resistance measured in Ringer was 5–10 MΩ.

All recordings were performed in the whole-cell configuration, using either a Cairn Optopatch or a custom-built amplifier (Cairn Research Faversham, United Kingdom).

Currents were low-pass filtered (typical cut-off frequency 0.5–1.5 KHz) and digitized at 16-bits with sampling frequencies ranging from 3 to 10 KHz using an analog-digital interface (Data Translation DT9834; Marlboro, MA) which also served to apply stimuli. Software for acquisition, stimulation, and processing of traces was developed in-house. Recording electrodes were positioned using Huxley-type micromanipulators (Custom Medical Research Equipment, Blackwood, NJ), while cells were visualized with a 40× objective.

### Chemical Stimulation

Cholinergic stimulation employed carbachol (Alfa Aesar, Ward Hill, MA), an agent that has been amply utilized with HEK-293 cells (Luo et al., 2008) as well as with SHsy5y cells (Lambert and Nahorski, 1990; Kelly et al., 1996; Larsson et al., 1998; Van Acker et al., 2002). In initial tests, a defined volume (20–200 μl) of relatively concentrated carbachol solution (100 μM, made by diluting a 10 mM stock into standard external solution) was applied to the chamber (vol. ≈600 μl). Subsequently, rapid, local solution changes were implemented using a puffer pipette positioned near the cell (≈20–30 μm) with the aid of a micromanipulator, either manual (Thorlabs, Newton, NJ) or motorized (Eppendorf Patchman, Hamburg, Germany). An air pump, pressure regulator, and pressure gauge, together with a solenoid-controlled valve permitted the delivery of controlled pressure pulses under computer control. Carbachol concentration in the puffer pipette was 1–5 μM. This approach was also used with other pharmacological agents that can be applied extracellularly. The PLC activator m-3M3FBS and its inert analog o-3MFBS were obtained from Tocris (Tocris Bioscience, Bristol, United Kingdom) and maintained as a 2 mM stock solution in DMSO. The PLC inhibitor U-73122 and its inert analog U-73343 purchased from Enzo (Farmingdale, NY) were dissolved as a 5 mM stock solution in dimethyl sulfoxide (DMSO). The stock solutions were then diluted in Ringer to the desired final concentration; the final DMSO concentration applied with the various drugs never exceeded 0.75%. Pilot measurements with 1% DMSO showed no effect on membrane currents nor on calcium fluorescence. In some experiments, GTP in the internal solution was replaced with 100 μM of the poorly hydrolyzable analog GTP-γ-S (Sigma), and dialyzed *via* the patch pipette. Lithium was introduced in the external solution by equi-molar replacement of Na, and in most cases was applied by superfusion of the entire chamber.

### Calcium Fluorescence Recordings

Changes in cytosolic calcium concentration were monitored in individual cells using the fluorescent indicator Fluo-4 (Invitrogen—Molecular Probes). Cells were pre-loaded by incubation with Fluo-4AM (2–5 μM for 40 min at 37°C in the presence of 0.5% Pluronic F127 and DMSO), followed by a wash in Ringer. A 470 nm LED (Thorlabs) was used for epi-illumination in most experiments; in some early trials, a 75 W Xenon arc lamp (PTI, So. Brunswick, NJ) was used instead, with a heat filter and an electromechanical shutter (Uniblitz Vincent Associates, Rochester, NY) coupled to the epi-fluorescence port of the microscope *via* a liquid light guide (Oriel Corporation, Stratford, CT). The illuminator output was filtered by a band-pass interference filter (Chroma, 480/40 nm; Chroma Technology, Bellows Falls, VT), and bounced by a 505 nm dichroic reflector in the microscope turret. An adjustable mask (Nikon) was placed at the image plane in the emission pathway and permitted to delimit the area of light collection. The light was split by a second dichroic reflector (570 nm), so that fluorescence was diverted to a photomultiplier (PMT, Hammamatsu R4220 PHA; Bridgewater, NJ) preceded by an electromechanical shutter (Uniblitz Vincent Associates) and a barrier filter (520–560 nm, Chroma). Long-wavelength light from a transmitted-light illuminator was instead focused onto the sensor of a CCD camera, in order to visualize the field of the collected light, when the adjustable mask was sized and positioned around the target cell to limit parasitic light. The PMT tube was operated at 640–700 V and its output passed through a window discriminator (F-100T, Advanced Research Instruments, Boulder, CO) which fed a photon counter (PRM-100, Advanced Research Instruments). A signal proportional to the counts accumulated in successive intervals (10–100 ms) was recorded by the computer.

The Ca responses of HEK cells stimulated pharmacologically by whole-bath application of the agonist were generally quite slow, necessitating a prolonged interval of data collection that could extend for up to 3 min. In those cases, a pulsed protocol was implemented (Gomez and Nasi, 2009) with the aim of reducing light exposure, and thus minimizing fluorophore bleaching and cell damage. To this end, the epi-illuminator was cyclically turned on every 3 s for 30 ms, and the fluorescence was monitored during the mid-20 ms of each pulse. This reduced irradiation by 99% without sacrificing the temporal resolution of the Ca signal. In experiments with ShSy5y cells, where the response to the cholinergic agonist applied locally was more rapid, continuous epi-illumination was utilized instead, reducing the fluorescence data-collection interval to 25 s. Fluo-4 has a nominal Kd for Ca of 335 nM according to the manufacturer, appropriate to report the range of concentration changes described in the cells that were used (see Ridley et al., 2002). Because this indicator is not ratio-metric, no absolute quantitation of Ca concentration is feasible, and data are reported in terms of relative fluorescence changes (ΔF/F).

## Results

The first goal was to determine whether the upregulation of the PLC pathway that had been observed in photoreceptors of the lancelet after iso-molar Na replacement with Li, can be reproduced in more standard preparations of mammalian cells, and also obtains at much lower doses, which may be of pharmacological relevance. To this end, HEK-293 cells were initially selected as a convenient model system: microarray analysis (Atwood et al., 2011) had previously revealed significant mRNA levels for the core elements of the phosphoinositoid cascade: G_q_ and PLC-β; in addition, mRNA for diverse metabotropic receptors was found. Especially relevant to the present purposes is the M3 variant of the muscarinic ACh receptor, because of its preferential coupling to G_q_ (Offermanns et al., 1994; Blin et al., 1995). Functional studies in cultured populations of wild-type HEK-293 loaded with Ca indicators had demonstrated that the application of cholinergic agonists induces an increase in fluorescence, attributed to the activation of M3 AChRs (Luo et al., 2008). The lack of reported nicotinic receptors and voltage-dependent Ca influx in HEK-293 cells reduces confounding regarding the mechanisms underlying Ca mobilization.

**Figure 1:**
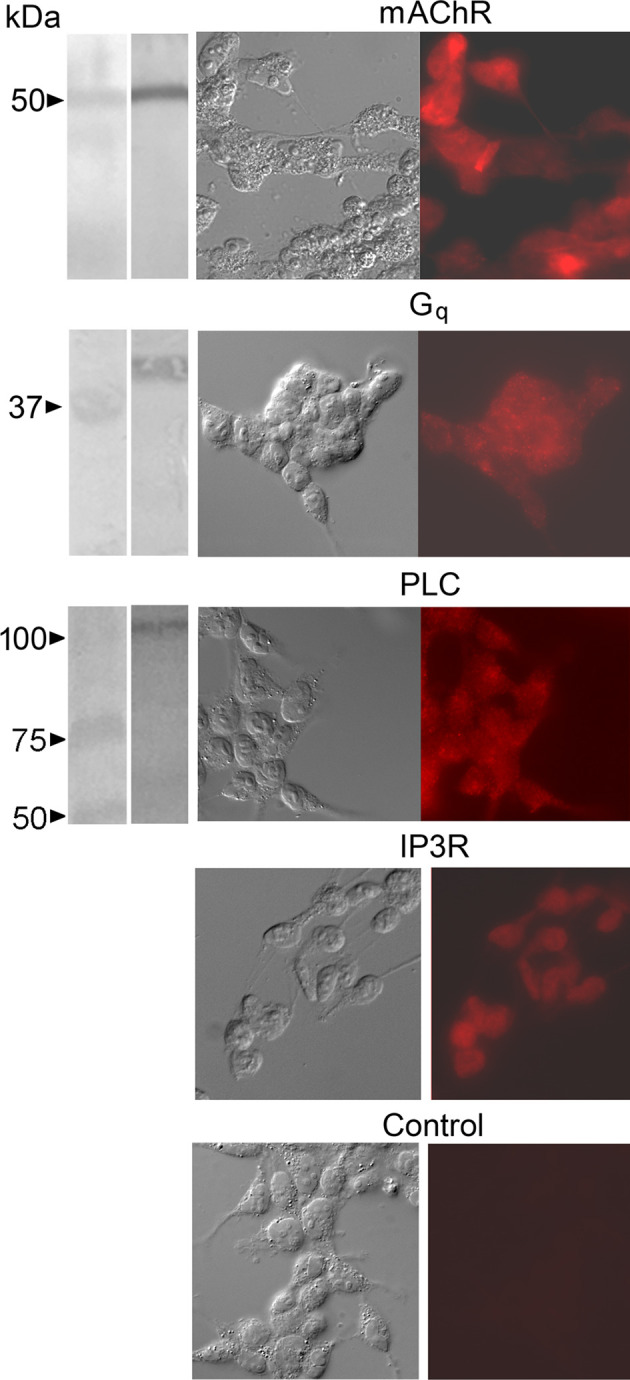
Immuno-detection of key signaling proteins of the PLC pathway coupled to muscarinic receptors in HEK-293 cells. Antibodies against the isoform M3-mAChR, the alpha subunit of the heterotrimeric G_q_ protein, PLC-β1, and the IP_3_ receptor type I were used. *Left column*: in Western blots a single band of the appropriate apparent molecular mass was obtained in each case (except for the IP_3_R, which appeared not to penetrate the separating gel even at the lowest acrylamide concentration used). The second column shows DIC micrographs of cultured cells, whereas fluorescence images of the same fields (*third column*) revealed consistent labeling (including the IP_3_R). Control cells only exposed to secondary antibodies remained unlabeled.

As an initial step, antibodies were used to corroborate the expression of relevant signaling proteins in cell lysates and in cell cultures, complementing the published data on mRNA. Targeted molecules included the muscarinic receptor M3-mAChR, the alpha subunit of the heterotrimeric G_q_ protein, PLC-β1, and the IP_3_ receptor type I. As shown in Figure 1, Western blots (which did not include IP_3_R, because even at 6% acrylamide gel penetration was dubious) yielded positive results—confirming the apparent molecular mass of the targets—as did immunofluorescence assays in cultured cells. Control cells only exposed to secondary antibodies remained unlabeled.

**Figure 2:**
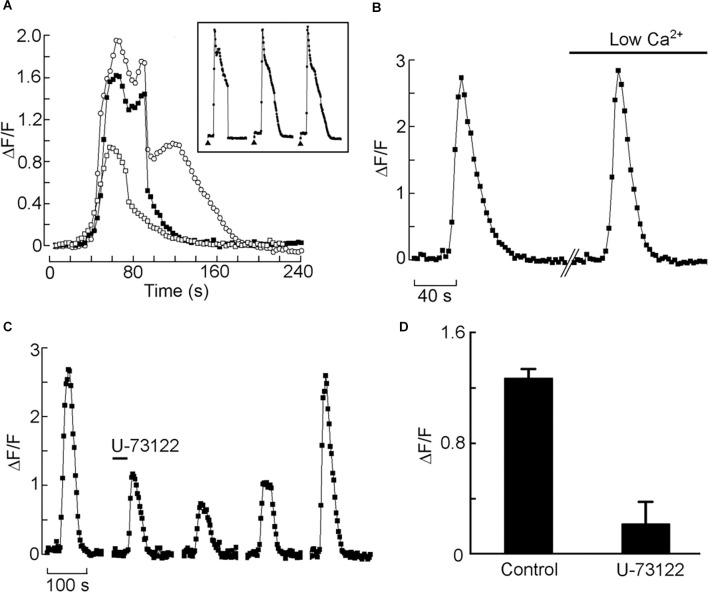
Cholinergic stimulation of HEK 293 cells induces mobilization of Ca^2+^ from intracellular stores, mediated by the PLC pathway. **(A)** Bath application of charbachol produced a distinct increase in fluorescence in a single Fluo-4-loaded cell; the Ca^2+^ signal increased with the amount of agonist applied to the bath. The three traces were recorded in the same cell (open squares: 20 μl; filled squares: 50 μl; open circles: 200 μl of 100 μM carbachol applied to the chamber inlet). The amplitude and time course of the response was highly reproducible with repeated application of the same dose, spaced 10 min apart *(inset)*. **(B)** The carbachol-induced mobilization of Ca is impervious to the removal of most extracellular Ca. The two responses were elicited in an individual cell in standard Ringer solution, and after reducing Ca to 100 μM, with no appreciable change. **(C)** Application of the PLC antagonist U-73122 (10 μM) markedly decreased the Ca response to carbachol, the effect being slowly reversible. **(D)** Pooled data for four cells, showing the degree of inhibition at the antagonist dose utilized.

Next, we set up a test to monitor Ca mobilization and assess Li effect in individual HEK-293 cells, similar to that described by Peinado et al. (2015). In Figure 2A the effects of applying carbachol directly to the bath are shown. Three different doses were tested, and a rest period of 10 min was interposed between applications. After a latency of several seconds—attributable partly to the perfusion and diffusion delay—the fluorescence intensity measured during the repetitive light pulses rose substantially, indicating an increase in intracellular Ca. The amplitude of the Ca transient was dose-dependent; at the higher agonist doses, a secondary “hump” appeared, and the time course became more prolonged, attaining a total response duration in excess of 2 min. The Ca response proved quite reproducible within cells, both in amplitude and time course (*Inset*). Most cells tested responded in a similar way (*n* > 20). We next ascertained the source of the Ca increase: Figure 2B shows the Ca response evoked by repeated application of the same dose of carbachol, first in control extracellular solution, and then 10 min after switching to an extracellular solution in which Ca was reduced 25-fold (from 2.5 mM to 100 μM; prolonged exposure to completely Ca-free solutions tended to compromise cell viability). The amplitude of the two fluorescence signals, in this case, was essentially indistinguishable (average reduction 19% ± 12.3 SD, *n* = 3), indicating that the Ca increase is due primarily to release from intracellular stores. Such response is dependent on PLC activity, as confirmed by the effects of the PLC inhibitor U-73122: in Figure 2C, repeated cholinergic stimulation was administered at 10 min intervals; before the second application of carbachol, the U-73122 was introduced in the recording chamber (20 μl, 50 μM). The amplitude of the Ca-mobilization signal was greatly depressed, the effects persisting for many min. Prolonged wash (>20 min) eventually restored the full amplitude of the Ca signal. The average reduction of the fluorescence signal after treatment with U-73122 was 76.8% ± 19.1 SD (*n* = 4; Figure 2D). The inert analog U-73343 was ineffective.

**Figure 3:**
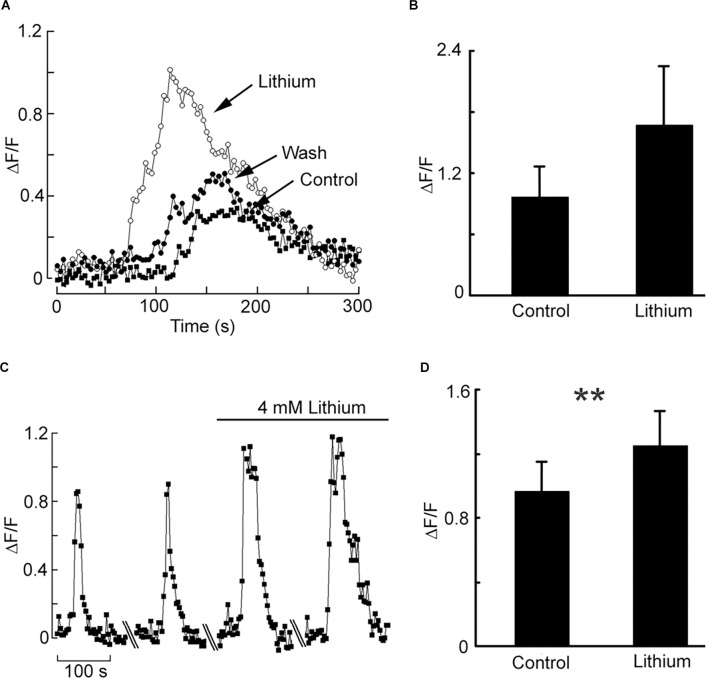
Effect of acute lithium exposure on the release of internal calcium in HEK cells. **(A)** A cell loaded with Fluo-4 was repeatedly stimulated with carbachol at intervals of 10 min. The first stimulus was applied in a normal extracellular solution; immediately afterward the perfusion was switched to a solution in which Na was replaced with Li. The Ca response increased conspicuously. Returning to the control solution re-established the initial response size. **(B)** Pooled data summarizing the magnitude of the potentiation upon iso-molar replacement of Na with Li. **(C)** Effect of 4 mM Li on the muscarinic Ca-mobilization response. After two control responses of similar amplitude and time course, the chamber was superfused with Li-containing solution. Diagonal lines mark a 10 min break between trials.The size of the carbachol-elicited response increased appreciably. **(D)** Bar-graph of the pooled data, showing the peak relative Ca increase in the two conditions. The double asterisk indicates statistically significant differences at *p* < 0.01.

Having documented a robust muscarinic-induced mobilization of internal Ca in HEK-293, we tested the effect of iso-osmotic Na replacement with Li. Figure 3A shows a representative example: in the presence of Li the amplitude of the Ca-fluorescence signal was dramatically increased, the effect being reversible. Figure 3B shows the data pooled for four cells; the ratio of peak ΔF/F in the presence of lithium *vs*. control was 1.71 ± 0.24 SD. Therefore, the upregulation by lithium of internal Ca mobilization initially observed in a primitive chordate is reproducible in a human cell line; nonetheless, it was obtained at Li concentrations that could at most be of toxicological relevance. An obvious question is whether such modulatory effect persists at lower Li concentrations, eventually aiming at therapeutic levels. To this end, we first briefly examined the outcome of a 10-fold decrease, to 14 mM. In two cells tested, the potentiation of carbachol-induced liberation of Ca was still clearly observed, though less pronounced (the Ca-fluorescence signal increased by 25% and 17%, respectively). Encouraged by this result, we assessed in a more systematic manner the effect of a further 3.5-fold reduction, to 4 mM. Figure 3C illustrates an experiment in which Ca was monitored with two applications of carbachol spaced 10 min apart, followed by a solution change to 4 mM Li and two more stimulations. Again, the Ca response was enhanced in the presence of Li: the average ΔF/F for a group of nine cells increased from 0.96 ± 0.57 SD to 1.25 ± 0.65 SD in the presence of 4 mM lithium (Figure 3D). The difference was statistically significant (Wilcoxon paired samples test, *p* < 0.01). The change to lithium-containing solution by itself produced no change in basal fluorescence.

**Figure 4:**
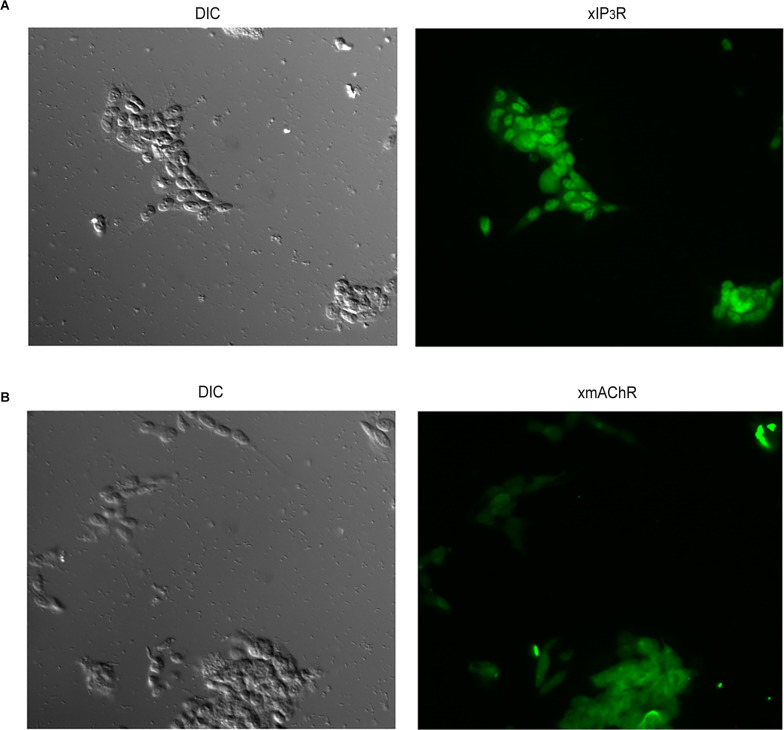
Verification of expression of proteins comprising the receptor and effector ends of the phosphoinositide signaling in SHSY5Y neuroblastoma cells. Antibodies against the muscarinic receptor M3-mAChR **(A)** and the IP_3_ receptor type I **(B)** were used in immunofluorescence assays, yielding distinct staining in both cases. Control cells only exposed to secondary antibodies remained unlabeled (*not shown)*.

We next sought to extend these results to a neuronal context, while retaining the convenience of a cultured cell line, and also ascertain Li effects on electrical signaling. SHsy5y is a line of human neuroblastoma that shares important features with primary neurons, including the expression of voltage-dependent channels selective for Na^+^ (Toselli et al., 1996; Vetter et al., 2012), K^+^ (Tosetti et al., 1998), and Ca^2+^ (Toselli et al., 1991; Sousa et al., 2013), and can be induced to generate action potentials (Toselli et al., 1996; Sonnier et al., 2003). In addition, SHsy5y cells express key molecules for vesicle exocytosis and are capable of Ca-dependent release of noradrenaline (Goodall et al., 1997; Mathieu et al., 2010). Of particular relevance to the present study, the expression of endogenous muscarinic receptors coupled to the hydrolysis of phosphatidylinositol has been reported (Fisher and Heacock, 1988; Mei et al., 1988); moreover, carbachol administration caused an increase in Ca fluorescence in cell suspensions, and an increase in IP_3_ (Lambert and Nahorski, 1990). Before assessing the reproducibility of the Li effects documented in HEK293 cells, we briefly verified by immunocytochemistry that the two proteins representing the end-points of the pathway of interest (that is, muscarinic receptors and IP_3_Rs) exhibited a robust expression in our cultures of SHsy5y cells (Figure 4).

**Figure 5:**
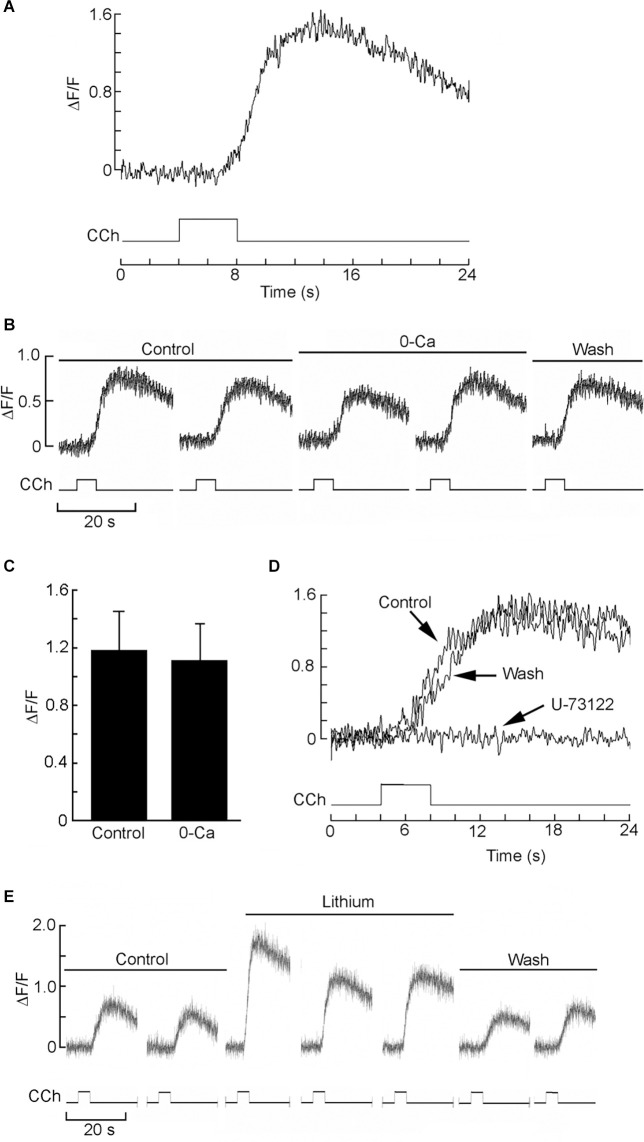
Mobilization of internal Ca in SHSY5Y cells and its upregulation by lithium. **(A)** Example of fluorescence increase in a Fluo-4-loaded SHSY5Y cell, induced by brief (4 s) ejection of 1 μM carbachol from a locally positioned puffer pipette. **(B)** Persistence of the Ca signal upon removal of Ca from the extracellular solution. **(C)** Bar-graph of pooled data, demonstrating the resilience of the charbacol-induced Ca mobilization upon lowering external calcium. **(D)** Elimination of the carbachol-induced increase in Ca upon exposure to the PLC antagonist U-73122 (50 μM). The suppression of the response was virtually total, and recovered near the original amplitude only after extensive wash. **(E)** Lithium (10 mM) augmented the muscarinic Ca-mobilization response, in a reversible manner.

Next, we corroborated the mobilization of intracellular Ca^2+^ induced by cholinergic stimulation in single SHSY5Y cells. For better temporal control, we turned to local application of carbachol by a puffer pipette which was lowered close to the target cells (≈30 μm). Figure 5A shows the increase in Ca fluorescence in a Fluo-4-loaded cell in response to 1 μM carbachol (*n* = 15). However, because these cells can express voltage-dependent Ca-channels (Toselli et al., 1991; Sousa et al., 2013) and V_m_ was not clamped, one must assess potential contributions by a Ca influx (Dajas-Bailador et al., 2002). In addition, endogenous nicotinic receptors have also been reported in SHSY5Y cells (Gould et al., 1992; Lukas et al., 1993), and a small entry of Ca attributed to the α7 subtype has been noted (Dajas-Bailador et al., 2002). Figure 5B shows that the calcium fluorescence signal was largely unaffected by the removal of extracellular Ca. On average, the reduction was only 6% (*n* = 5, see Figure 5C), indicating that the observed Ca mobilization can be ascribed to release from internal stores. Furthermore, Figure 5D demonstrates that the PLC inhibitor U-73122 virtually abolished the carbachol-triggered response (concentrations of either 10 or 50 μM were tried; *n* = 6); like in the case of HEK-293 cells, the suppression was slowly reversible, requiring a wash period in excess of 15–20 min. Next, we tested whether the PLC-dependent Ca-mobilization in the SHsy5y cell line is affected by Li. Figure 5E shows that acute exposure to 10 mM Li reversibly enhanced the carbachol-evoked Ca-fluorescence signal; on average, ΔF/F increased by 58%, the effect being statistically significant (Wilcoxon test for paired samples *p* = 0.016, *n* = 7). Taken together, the results fully replicate in this neuronal cell line the upregulation by lithium of the PLC-dependent Ca mobilization observed in HEK-293 cells.

### Characterization of Electrical Muscarinic Response

**Figure 6:**
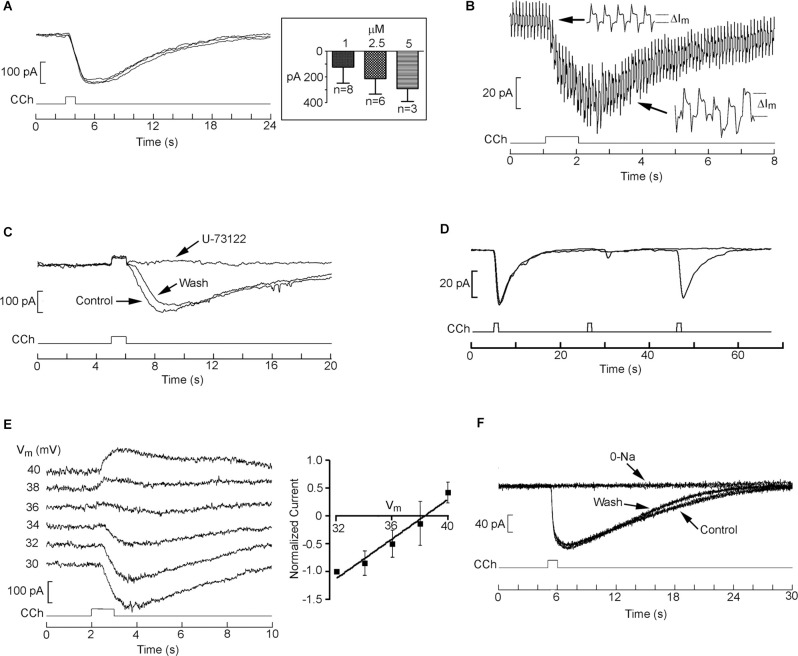
Whole-cell clamp recordings of cholinergic currents in SHSY5Y cells. **(A)** Repetitive 1-s puffer-pipette application of 1 μM carbachol evoked an inward current with highly reproducible amplitude and time course (holding potential −70 mV). *Inset*: dose-dependency of the peak amplitude of the current. **(B)** An increase in membrane conductance underlies the carbachol-elicited current. The holding potential was perturbed with a cyclic rectangular command 10 mV in amplitude, 10 Hz, 50% duty-cycle, causing repetitive transitions in the current trace. During the carbachol-triggered inward current, the size of the current jumps increased substantially (*insets with time-expanded samples*), indicating a drop in the value of the membrane resistance. **(C)** The cholinergic current is mostly due to muscarinic receptors tapping onto the PLC pathway: the response to carbachol was eliminated in a reversible manner upon application of U-73122 to the bath. **(D)** Double-pulse experiment to ascertain the recovery time of the muscarinic current after 1-s stimulation with 1 μm carbachol. Pulse pairs were spaced 3 min apart. Whereas with an inter-stimulus interval of 20 s the second response remained greatly attenuated, increasing the delay to 40 s resulted in a practically complete recovery of response amplitude. **(E)** Reversal potential of the muscarinic current. A cell was voltage clamped at −70 mV and repeatedly stimulated with puffer-application of carbachol (1 s, 1 μM). Several seconds before each pharmacological stimulus, membrane voltage was stepped to different depolarized values, as indicated. At V_m_ > 36 mV the carbachol-evoked current became outwardly directed. *Inset*: I-V relation, least-square fitted to a linear relation. **(F)** Disappearance of the muscarinic response upon replacement of extracellular Na with Tris.

While the effect of Li on Ca release is robust, the therapeutic impact of this agent must also be reflected in some alteration of the electrical activity of target nerve cells. We, therefore, examined membrane currents evoked by cholinergic stimulation in SHsy5y cells. Figure 6A shows three superimposed traces of inward current triggered by a 1 s puffer application of 1 μM carbachol spaced 3 min apart; the amplitude and time course were highly reproducible. Concentration-dependence is shown in the *inset* and served the purpose of ensuring that the stimulation conditions adopted were not saturating, which may interfere with the detection of modulatory effects. This response is due to the opening of ion channels, as determined by concomitantly measuring membrane resistance *via* the application of repetitive perturbations of the holding voltage: as illustrated in Figure 6B, the amplitude of the resulting current jumps (*see enlarged insets*) increased during the cholinergic response, reporting a decrease in input resistance from 1.4 ± 0.06 to 0.55 ± 0.03 GΩ. Figure 6C shows that bath application of 10 μM U-73122 fully suppressed the carbachol-induced inward current in a partially reversible manner (*n* = 4), so that it can be ascribed to PLC-coupled muscarinic receptors. Because subsequent measurements called for multiple treatments and stimulations, it was paramount to optimize the limited time-window of cell viability during whole-cell clamp recording—which is far shorter than that of the less invasive Ca-fluorescence assays. To gauge the maximum frequency of stimulus repetition that could be confidently employed, we assessed the recovery from desensitization of the muscarinic response in a double-pulse experiment. Figure 6D shows paired applications of carbachol separated by 20 or 40 s; trials were in turn spaced 3 min apart. Whereas the shorter inter-pulse interval leaves the second response substantially attenuated, with 40 s the recovery was practically complete. We thus adopted a 1-min wait-time as standard. To characterize the basic ionic properties of the response, cholinergic stimulation was applied repetitively as the holding potential was systematically depolarized at 2 mV increments. Figure 6E shows the reversal of the current; a plot of the average normalized currents for a group of cells is shown on the right. The mean reversal potential was 38.5 ± 1.3 mV (*n* = 5). Replacement of extracellular Na with Tris essentially abolished the inward current measured at the standard holding potential of −70 mV, the effect being fully reversible (Figure 6F, *n* = 6). However, the carbachol-activated conductance cannot be purely sodium-selective, as the Nernst potential for Na under those conditions was calculated at + 66 mV. Some participation of K ions (E_K_ = −82 mV) and/or a non-negligible permeation of Tris likely explain the observations.

### Modulation of Electrical Responses by Li

**Figure 7:**
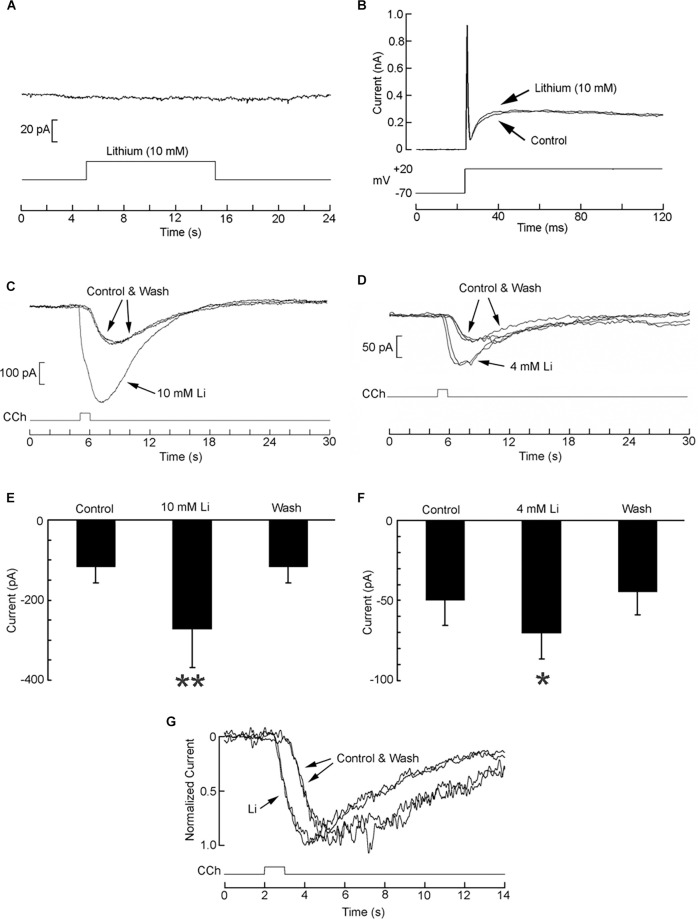
Potentiation of the muscarinic current by lithium. **(A)** Local application of Li in the absence of cholinergic stimulation failed to alter the holding current (Vm = −70 mV). **(B)** Depolarization of the membrane potential from −70 to +20 mV triggered voltage-dependent active currents that were unaffected by superfusion of lithium. **(C)** A SHSY5Y cell under voltage-clamp at −70 mV was stimulated with 1 μM carbachol locally applied by puffer pipette first in standard extracellular solution, then upon perfusing the chamber with 10 mM Li (replacing Na), and again upon returning to control conditions. The muscarininc current was greatly enhanced, in a fully reversible way. **(D)** Enhancement of the carbachol-elicited current upon exposure to 4 mM Li. A consistent, reversible effect was again obtained. **(E,F)** Pooled data comparing average peak amplitude of the muscarinic current in control solution *vs*. lithium; data obtained in seven cells in each experiment. **(G)** Decrease of the response latency in the presence of 4 mM Li. Current traces were normalized with respect to the peak amplitude. *p < 0.05; **p < 0.01.

The muscarinic current was examined in the presence of lithium. The initial concentration tested was 10 mM. We first ascertained that in the absence of receptor activation lithium produced no effect on membrane currents: in Figure 7A a cell was voltage-clamped at −70 mV and lithium was locally applied by a puffer pipette for 10 s; the holding current was unaffected (*n* = 3). In panel B a cell was stimulated with a depolarizing pulse to +20 mV in control conditions and in the presence of Li. Again, no significant change occurred in the voltage-activated currents (*n* = 3). By contrast, Figure 7C shows that the current evoked by puffer application of 1 μM carbachol was strongly and reversibly enhanced with bath perfusion with Li. Panel E of the same figure shows the pooled data corresponding to the peak amplitude of the current in seven cells. The relative increase in current amplitude was 116.8 ± 50.7% (*p* = 0.004, Wilcoxon test for paired samples). Given the strong effect obtained, a lower concentration of Li was assessed. Figures 7D,F show that the potentiation upon exposure to 4 mM Li remained conspicuous (average 61.6 ± 41.1%), and statistically significant (*p* = 0.032). The enhancement of the muscarinic current in the presence of Li was accompanied by a shortening of the latency: Panel G of Figure 7 shows normalized, superimposed current traces recorded before, during, and after exposure to 4 mM Li. The latency was reduced on an average by 539 ± 156 ms.

### Site of Action

The robust regulatory effects of lithium, documented both in electrical responses and Ca-mobilization mediated by stimulation of the PLC signaling pathway, naturally raise the question of possible site(s) of action. A general strategy to address this question entails directly stimulating the cascade at various links, bypassing the receptor. One would anticipate that as long as Li acts at some step downstream of the link that was tapped, its modulatory capability will remain manifest, whereas if its site of action is upstream the effect will be lost. Observations of this nature can therefore help delimit—or at least narrow down—the target of the observed action of lithium.

**Figure 8:**
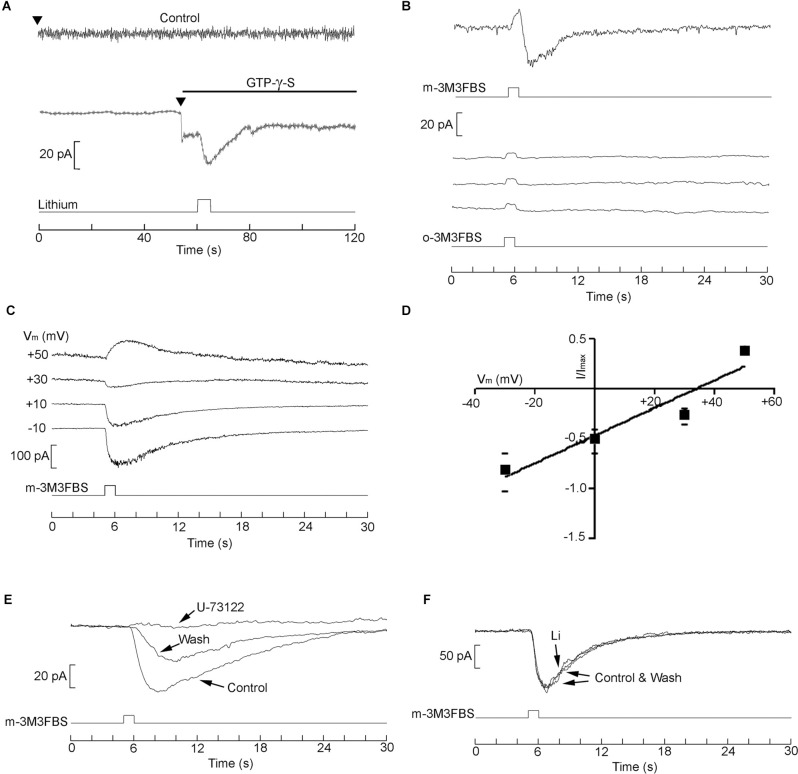
Delimitation of the site of action of Li. **(A)** membrane current evoked by intracellular perfusion with GTP-γ-S. *Top current trace*: when the patch pipette contained standard internal solution, the holding current (at V_m_ = −70 mV) remained stable upon accessing the cell interior. Local perfusion with 10 mM lithium with a puffer pipette (*indicated in the bottom trace*) produced no change in current. By contrast, when the pipette contained GTP-γ-S, an inward current rapidly developed upon initiating intracellular perfusion (*arrowhead*), and subsequent local application of Li enhanced its amplitude. **(B)** Activation of membrane current by direct stimulation with the PLC activator m-3M3FBS (2.5 μM), compared to the inert analog (o-3M3FBS; three repetitions). **(C,D)** Test of the reversal potential and I-V relation of the current evoked by m-3M3FBS. **(E)** Susceptibility of the m-3M3FBS-induced response to the PLC inhibitor U-73122. **(F)** The response activated by direct stimulation of the PLC proved insensitive to lithium exposure.

We applied GTP-γ-S by internal dialysis to activate the G-protein and examined the effects of exposure to lithium. The top trace in Figure 8A shows that in the absence of GTP-γ-S the holding current remained stable upon attaining the whole-cell configuration (*arrowhead*). Moreover, subsequent puffer application of 10 mM Li (*bottom trace*) produced no effect. By contrast, when the patch pipette contained GTP-γ-S (*second trace*), an inward current developed upon rupturing the patch to access the cell interior; when lithium was locally applied, the amplitude of the inward current was potentiated (*n* = 6).This indicates that the effect of lithium must occur at the level of the G-protein or further downstream, and does not implicate the receptor. We next directly stimulated pharmacologically the phospholipase C. The substance m-3M3FBS is a membrane-permeable activator of PLC- β (Bae et al., 2003). Application of 2.5 μM m-3M3FBS to SHsy5y cells by puffer pipette evoked, shortly following the 1 s pressure pulse, an inward current (Figure 8B, top trace; *n* = 4). By contrast, repetitive stimulation with the inert form o-3M3FBS (*middle traces of* Figure 8B) only produced the stimulus artifact during activation of the solenoid valve, but no physiological response. To strengthen the contention that the current evoked by m-3M3FBS is akin to that triggered by muscarinic stimulation, responses were measured while membrane potential was held at different levels (Figure 8C). The current reverted at a similar voltage (32.7 ± 6.8 mV, *n* = 4) as the muscarinic current, as illustrated by the I-V curve shown in Panel D. Because there has been a report of m-3M3FBS effects that do not involve PLC (Krjukova et al., 2004), which could skew data interpretation, we also tested the antagonist U-73122. As shown in Figure 8E, the response was abolished in a partially reversible manner (*n* = 3) confirming that it was mediated by PLC. At that point, we evaluated the effect of lithium on such response but failed to detect any sign of potentiation with bath application of lithium at either 4 mM (*n* = 4) or 10 mM (*n* = 6). Panel F of Figure 8 illustrates an example, in which the traces of m-3M3FBS-evoked current recorded in control conditions and in the presence of 10 mM Li are superimposable. The data, therefore, indicate that the observed Li modulation arises upstream of the activated PLC, and implicate an effect on G_q_ or its interaction with its target.

## Discussion

The molecular targets and specific mechanisms of action of lithium have long been a puzzle, and a wide spectrum of candidates has been implicated. Much of the work in this field has tackled the problem primarily from a biochemical perspective; by contrast, little is known about lithium-induced alterations of specific physiological responses at the cellular level. In view of the plethora of possible Li effects—likely to occur concomitantly—it is critical to fill such gap because changes in neuronal activity are difficult to infer solely on the basis of alterations in various biochemical parameters. In this work, the modulatory influence of acute lithium exposure on the PLC signaling pathway was assessed in two different mammalian cell lines, by monitoring internal Ca mobilization and membrane currents triggered by activation of metabotropic receptors: both responses are of paramount importance for the functioning of neuronal circuits, as they are implicated in ubiquitous processes of inter-cellular communication in the brain. The data garnered with Li levels in the low millimolar range reveal a robust, highly reproducible potentiation of PLC-dependent release of Ca from intracellular stores, as well as an augmentation of the amplitude of membrane currents that are triggered by G_q_ protein-linked receptors.

Two important, related issues must be addressed in order to appraise the possible relevance of the present observations to the clinical use of Li:

### Concentrations of Lithium Utilized

Standard effective Li concentration in plasma for therapeutic purposes—without incurring toxic side-effects—is around 0.6–1.2 mM (Timmer and Sands, 1999), clearly lower than the concentrations employed in the present study. However, serum levels of Li typically refer to “trough” values (12 h post-administration; Lauritsen et al., 1981). Actual time-point measurements indicate wide fluctuations, depending on the frequency of intake, which can amply exceed 100% (Amdisen, 1973, 1978; Bergner et al., 1973; Lauritsen et al., 1981). That being the case, the lowest concentrations explored in the present study (4 mM)—while higher than those adopted in clinical usage and undoubtedly toxic if applied in a sustained manner—may not diverge too much with respect to peak serum levels that can be attained, albeit transiently, in a therapeutic context. Of course, a more relevant parameter is the concentration of Li bathing the target cells in the brain: estimated values for the CSF are generally lower, according to NMR measurements *in vivo*, the ratio CSF:plasma being on the order of 0.4–0.8 in most cases, but ratios >1 (*e.g.*, up to 1.3) have also been reported (reviewed by Soares et al., 2000). Like in serum, Li concentrations in brain tissue also experience temporal fluctuations (Plenge et al., 1994). But, in view of its intracellular site(s) of action, an even more pertinent issue is the internalization of lithium, which is slow: with the exposure that in the present study only lasted for minutes, internal levels attained may be more in line with those reached under prolonged exposure at lower doses. Finally, to complicate the matter further, spatial variations enter the picture prominently, as non-homogeneities and significant Li accumulation in discrete regions of the brain has been described (Lee et al., 2012; Smith et al., 2018). Therefore, much remains to be clarified about therapeutic Li levels to which the relevant target cells—which in turn are not known—are effectively exposed, and therefore the concentrations we used cannot be dismissed as irrelevant.

### Acute vs. Chronic Li

The second issue relates to the fact that we examined the impact of acute exposure, whereas Li therapy notoriously entails a prolonged treatment, with the full spectrum of beneficial effects becoming manifest only after several days. Here too, two separate pertinent aspects must be taken into account. In the first place, one must consider factors that reflect the kinetics of Li bio-availability when the application is systemic—as is the case in clinical use: various studies have shown that plasma levels increase slowly, reaching a plateau regime in a matter of several days, and the attainment of such asymptotic behavior in the brain is apparently further delayed with respect to serum (Renshaw and Wicklund, 1988). Therefore, at least for some variables, the need for prolonged exposure to Li may be partly a reflection of the slow build-up of serum levels, and can help explain the ineffectiveness of briefer exposure (for example, see Colin et al., 1991; Sourial-Bassillious et al., 2009). In support of this notion, certain effects of Li that normally require prolonged systemic administration could also obtain acutely, provided that the application is implemented directly to the tissues/cells (Wang and Friedman, 1999; Farhy Tselnicker et al., 2014). Nonetheless, there are also effects of Li that genuinely require a more prolonged time span in order to become manifest, not simply because of sluggish attainment of the required concentrations. One instance concerns alterations in the turnover/expression levels of key target molecules: Colin et al. (1991) reported that rats exposed to chronic (4-week) treatment with therapeutic doses of Li suffered an increase in cerebral cortex expression levels of adenylate cyclase, with a concomitant decrease in G_i_ expression (other G-proteins being seemingly unaffected); the effect did not occur with shorter treatment (6 days). Likewise, Sourial-Bassillious et al. (2009) describe a marked reduction in the expression of mGluR5 receptors in hippocampal neurons, an effect that was not seen with short Li exposure. Other effects too develop slowly: it has been reported that irrespective of the expression level of Gα, there appears to be a change in receptor-stimulated GTP-binding that also becomes manifest only with prolonged Li treatment (Wang and Friedman, 1999). Finally, from the inositol-depletion hypothesis, one may surmise a negative effect of lithium on phosphoinositol signaling, whereas we documented a robust enhancement. Such depletion likely requires more prolonged exposure to lithium and could exert an influence on signaling when inositol supply is limited, to begin with. In this regard, it has been shown that profound differences exist across brain regions with regard to the ability of Li to reduce agonist-induced inositol polyphosphate accumulation (Kennedy et al., 1990). Disparate responses to Li can thus be anticipated depending on cells type and treatment duration. The rapid onset of the effects described here, arising from brief application of Li, most certainly concern direct regulation of signaling elements function, as modification of protein expression levels and substrate availability are not plausible in such time scale.

While it would seem reasonable to propose extending our observations to a prolonged treatment—akin to the clinical usage—exposing cultured cell lines “chronically” (many days) to Li in order to ascertain its effects would face difficulties. On the one hand, there is a loss of sensitivity inherent to switching from within-cell comparisons (easily implemented when the treatment is acute) to between-cell comparisons (namely, carrying out the measurements in cells chronically cultured in the presence of Li *vs*. cells from a different culture maintained under control conditions). Moreover, cell lines are constantly dividing, which introduces an uncertainty factor about the effective duration of Li exposure for any particular cell tested. Cultures of primary neurons would be more suitable for that purpose.

### Site of Action

It is noteworthy that, by exploiting the direct activation of the signaling cascade at sites downstream of the receptor, the site of action of Li could be narrowed down to G_q_ and its interaction with the PLC. *In vitro* biochemical demonstrations of the influence of lithium on heterotrimeric G-proteins have long been known: Avissar et al. (1988) and Wang and Friedman (1999) had reported that Li treatment—both acute *in vitro* and chronic *ex vivo—*interfered with agonist-induced activation of G_s_, G_i_ and G_o_ (but not G_q_), as assessed by GTP binding (but see Odagaki et al., 1997). On the other hand, the work of Farhy Tselnicker et al. (2014) indicates that Li de-stabilizes the interaction between Gα_i/o_ and Gβγ. The present results attest an upregulation of G_q_/PLC function; its mechanistic underpinnings remain to be investigated, but could conceivably turn out to be akin the facilitation of the G-protein activation cycle described by Farhy Tselnicker et al. (2014) in the case of G_o_.

### G_q_ Cascades in the Brain, That May Be Targeted by Li

Although we focused on the muscarinic response mediated by PLC-linked M3 receptors as a model to assess modulation by Li, the observations indicating a site of action downstream of the receptor raise the possibility that similar lithium effects may extend to other neurotransmitters and metabotropic receptors that tap into the same signaling pathway. The family of G_q_/PLC-linked receptors includes muscarinic receptors M1, 3, and 5, the glutamatergic receptors mGluR1 and 5, purinergic receptors P2Y1, 2, 4, 6, adrenergic α1 receptors (Wu et al., 1992), 5HT receptors sub-types 2a-c (McCorvy and Roth, 2015), among others. This hints at a very wide range of potential Li effects on synaptic communication, that could affect diverse brain structures: in mammals, phosphoinositide signaling—as gauged by IP_3_ receptor expression—includes Purkinje neurons of the cerebellum, the hippocampus CA1 layer, the olfactory bulb, the striatum, the septum, and others (Nakanishi et al., 1991). However, the scenario might be simplified if such Li action exhibited some selectivity with respect to the specific isoforms of the signaling molecules involved: beyond the canonical Gq and its sibling Gα11, PLC-linked G-protein also includes the more recently uncovered Gα15–16 (Amatruda et al., 1991; Offermanns and Simon, 1995)—being of special interest because it can mediate interaction with PLC of receptors that normally couple to other pathways. Likewise, diverse isoforms of PLCs activated by G-protein- exist (PLCβ1–4), and display characteristic distribution patterns (Watanabe et al., 1998). The present observations implicate G_q_ and—most likely—PLC β3, which are abundantly expressed in HEK-293 and in SHSY5Y cells, but future studies will need to scrutinize other combinations. Examination of lithium effects in various classes of isolated primary neurons that express different variants of the G-protein and the PLC will be especially informative. Should differential actions of Li be uncovered, it would narrow down brain structures and cell types that are susceptible, and help refine our understanding of its therapeutic function.

In summary, a robust modulatory influence of acute Li exposure was demonstrated, resulting in a specific short-term potentiation of well-defined physiological responses at the cellular level. This novel mechanism—whose site of action could be delineated—is likely to contribute to the complex pattern of therapeutic actions of Li, and may help understand the multiplicity of effects of this drug, that enable it to blunt both manic and depressive crises. The availability of detailed mechanistic information could possibly guide future efforts geared towards the development of improved pharmacological agents with greater specificity and reduced toxicity or side effects.

## Data Availability Statement

The raw data supporting the conclusions of this article will be made available by the authors, without undue reservation.

## Author Contributions

CS, ML, and SD performed experiments and data analysis. MG and EN contributed to conception and design of the study, supervised the project, analyzed data, and wrote the manuscript. All authors contributed to the article and approved the submitted version.

## Funding

This work was supported by *DIB-Universidad Nacional de Colombia*, grant Hermes No. 41821.

## Conflict of Interest

The authors declare that the research was conducted in the absence of any commercial or financial relationships that could be construed as a potential conflict of interest.

## Publisher’s Note

All claims expressed in this article are solely those of the authors and do not necessarily represent those of their affiliated organizations, or those of the publisher, the editors and the reviewers. Any product that may be evaluated in this article, or claim that may be made by its manufacturer, is not guaranteed or endorsed by the publisher.

## References

[B2] Allison J. H. Blisner M. E. (1976). Inhibition of the effect of lithium on brain inositol by atropine and scopolamine. Biochem. Biophys. Res. Commun. 68, 1332–1338. 10.1016/0006-291x(76)90342-9 1267780

[B1] Allison J. Bilsner M. Holland W. Hipps P. Sherman W. (1976). Increased brain myo-inositol 1-phosphate in lithium-treated rats. Biochem. Biophys. Res. Commun. 71, 664–670. 10.1016/0006-291x(76)90839-1 962945

[B3] Allison J. H. Stewart M. A. (1971). Reduced brain inositol in lithium-treated rats. Nature 233, 267–268. 10.1038/newbio233267a0 5288124

[B4] Amatruda T. T. Steele D. A. Slepak V. Z. Simon M. I. (1991). G alpha 16, a G protein alpha subunit specifically expressed in hematopoietic cells. Proc. Natl. Acad. Sci. U S A 88, 5587–5591. 10.1073/pnas.88.13.5587 1905813 PMC51922

[B5] Amdisen A. (1973). Serum lithium estimations. Br. Med. J. 2:240. 10.1136/bmj.2.5860.240-a 4700014 PMC1589379

[B6] Amdisen A. (1978). Clinical and serum-level monitoring in lithium therapy and lithium intoxication. J. Anal. Toxicol. 2, 193–202. 10.1093/jat/2.5.193

[B7] Atwood B. K. Lopez J. Wager-Miller J. Mackie K. Straiker A. (2011). Expression of G protein-coupled receptors and related proteins in HEK293, AtT20, BV2 and N18 cell lines as revealed by microarray analysis. BMC Genomics 12:14. 10.1186/1471-2164-12-14 21214938 PMC3024950

[B8] Avissar S. Schreiber G. Danon A. Belmaker R. H. (1988). Lithium inhibits adrenergic and cholinergic increases in GTP binding in rat cortex. Nature 331, 440–442. 10.1038/331440a0 3340189

[B9] Bae Y. S. Lee T. G. Park J. C. Hur J. H. Kim Y. Heo K.. (2003). Identification of a compound that directly stimulates phospholipase C activity. Mol. Pharmacol. 63, 1043–1050. 10.1124/mol.63.5.1043 12695532

[B10] Bergner P. E. Berniker K. Cooper T. B. Gradijan J. R. Simpson G. M. (1973). Lithium kinetics in man: effect of variation in dosage pattern. Br. J. Pharmacol. 49, 328–339. 10.1111/j.1476-5381.1973.tb08380.x 4793335 PMC1776389

[B11] Berridge M. J. Downes C. P. Hanley M. R. (1982). Lithium amplifies agonist-dependent phosphatidylinositol responses in brain and salivary glands. Biochem. J. 206, 587–595. 10.1042/bj2060587 7150264 PMC1158627

[B12] Beurel E. Grieco S. F. Jope R. S. (2015). Glycogen synthase kinase-3 (GSK3): regulation, actions and diseases. Pharmacol. Ther. 148, 114–131. 10.1016/j.pharmthera.2014.11.016 25435019 PMC4340754

[B13] Blin N. Yun J. Wess J. (1995). Mapping of single amino acid residues required for selective activation of G_q/11_ by the m3 muscarinic acetylcholine receptor. J. Biol. Chem. 270, 17741–17748. 10.1074/jbc.270.30.17741 7629074

[B14] Casebolt T. L. Jope R. S. (1987). Chronic lithium treatment reduces norepinephrine-stimulated inositol phospholipid hydrolysis in rat cortex. Eur. J. Pharmacol. 140, 245–246. 10.1016/0014-2999(87)90813-2 3666020

[B15] Casebolt T. L. Jope R. S. (1989). Long-term lithium treatment selectively reduces receptor-coupled inositol phosphoilipid hydrolysis in rat brain. Biol. Psychiatry 25, 329–340. 10.1016/0006-3223(89)90180-7 2536562

[B16] Colin S. F. Chang H.-C. Mollner S. Pfeuffer T. Reeds R. R. Duman R. S.. (1991). Chronic lithium regulates the expression of adenylate cyclase and Gi-protein a subunit in rat cerebral cortex. Proc. Natl. Acad. Sci. U S A 88, 10634–10637. 10.1073/pnas.88.23.10634 1720545 PMC52984

[B17] Dajas-Bailador F. A. Mogg A. J. Wonnacott S. (2002). Intracellular Ca^2+^ signals evoked by stimulation of nicotinic acetylcholine receptors in SH-SY5Y cells: contribution of voltage-operated Ca^2+^ channels and Ca^2+^ stores. J. Neurochem. 81, 606–614. 10.1046/j.1471-4159.2002.00846.x 12065669

[B18] Dixon J. F. Hokin L. E. (1998). Lithium acutely inhibits and chronically up-regulates and stabilizes glutamate uptake by presynaptic nerve endings in mouse cerebral cortex. Proc. Natl. Acad. Sci. U S A 95, 8363–8368. 10.1073/pnas.95.14.8363 9653192 PMC20981

[B19] Ebstein R. P. Hermoni M. Belmaker R. H. (1980). The effect of lithium on noradrenaline-induced cyclic AMP accumulation in rat brain: inhibition after chronic treatment and absence of supersensitivity. J. Pharmacol. Exp. Ther. 213, 161–167. 6244392

[B20] Farhy Tselnicker I. Tsemakhovich V. Rishal I. Kahanovitch U. Dessauer C. W. Dascal N. (2014). Dual regulation of G proteins and the G-protein-activated K^+^ channels by lithium. Proc. Natl. Acad. Sci. U S A 111, 5018–5023. 10.1073/pnas.1316425111 24639496 PMC3977261

[B21] Fisher S. K. Heacock A. M. (1988). A putative M3 muscarinic cholinergic receptor of high molecular weight couples to phosphoinositide hydrolysis in human SK-N-SH neuroblastoma cells. J. Neurochem. 50, 984–987. 10.1111/j.1471-4159.1988.tb03008.x 2828552

[B22] Godfrey P. P. McClue S. J. White A. M. Wood A. J. Grahame-Smith D. G. (1989). Subacute and chronic *in vivo* lithium treatment inhibits agonist- and sodium fluoride stimulated inositol phosphate production in rat cortex. J. Neurochem. 52, 498–505. 10.1111/j.1471-4159.1989.tb09148.x 2536074

[B23] Gomez M. Nasi E. (2009). Prolonged calcium influx after termination of light-induced Ca release in invertebrate photoreceptors. J. Gen. Physiol. 134, 177–189. 10.1085/jgp.200910214 19720959 PMC2737223

[B24] Goodall A. R. Danks K. Walker J. H. Ball S. G. Vaughan P. F. (1997). Occurrence of two types of secretory vesicles in the human neuroblastoma SH-SY5Y. J. Neurochem. 68, 1542–1552. 10.1046/j.1471-4159.1997.68041542.x 9084425

[B25] Gould J. Reeve H. L. Vaughan P. F. Peers C. (1992). Nicotinic acetylcholine receptors in human neuroblastoma (SH-SY5Y) Cells. Neurosci. Lett. 145, 201–204. 10.1016/0304-3940(92)90022-y 1465217

[B26] Hallcher L. M. Sherman W. R. (1980). The effects of lithium ion and other agents on the activity of myo-inositol-1-phosphatase from bovine brain. J. Biol. Chem. 255, 10896–10901. 10.1016/S0021-9258(19)70391-3 6253491

[B27] Inhorn R. C. Majerus P. W. (1987). Inositol polyphosphate 1-phosphatase from calf brain. Purification and inhibition by Li^+^, Ca^2+^ and Mn^2+^. J. Biol. Chem. 262, 15946–15952. 2824473

[B28] Jope R. (1979). Effects of lithium treatment *in vitro* and *in vivo* on acetylcholine metabolism in rat brain. J. Neurochem. 33, 487–495. 10.1111/j.1471-4159.1979.tb05179.x 469539

[B29] Jope R. S. (2003). Lithium and GSK-3: one inhibitor, two inhibitory actions, multiple outcomes. Trends Pharmacol. Sci. 24, 441–443. 10.1016/S0165-6147(03)00206-2 12967765

[B30] Jope R. S. Morrisett R. A. Snead C. (1986). Characterization of lithium potentiation of pilocarpine-induced status epilepticus in rats. Exp. Neurol. 91, 471–480. 10.1016/0014-4886(86)90045-2 3948956

[B31] Kelly J. F. Furukawa K. Barger S. W. Rengen M. R. Mark R. J. Blanc E. M.. (1996). Amyloid beta-peptide disrupts carbachol-induced muscarinic cholinergic signal transduction in cortical neurons. Proc. Natl. Acad. Sci. U S A 93, 6753–6758. 10.1073/pnas.93.13.6753 8692890 PMC39099

[B32] Kennedy E. D. Challiss R. A. Ragan C. I. Nahorski S. R. (1990). Reduced inositol polyphosphate accumulation and inositol supply induced by lithium in stimulated cerebral cortex slices. Biochem. J. 267, 781–786. 10.1042/bj2670781 2339988 PMC1131366

[B33] Krjukova J. Holmqvist T. Danis A. S. Akerman K. E. Kukkonen J. P. (2004). Phospholipase C activator m-3M3FBS affects Ca^2+^ homeostasis independently of phospholipase C activation. Br. J. Pharmacol. 143, 3–7. 10.1038/sj.bjp.0705911 15302681 PMC1575272

[B34] Lambert D. G. Nahorski S. R. (1990). Muscarinic-receptor-mediated changes in intracellular Ca^2+^ and inositol 1,4,5-trisphosphate mass in a human neuroblastoma cell line, SH-SY5Y. Biochem. J. 265, 555–562. 10.1042/bj2650555 2302186 PMC1136919

[B35] Larsson C. Thomas A. P. Hoek J. B. (1998). Carbachol-stimulated Ca^2+^ increase in single neuroblastoma SH-SY5Y cells: effects of ethanol. Alcohol. Clin. Exp. Res. 22, 637–645. 10.1111/j.1530-0277.1998.tb04305.x 9622444

[B36] Lauritsen B. J. Mellerup E. T. Plenge P. Rasmussen S. Vestergaard P. Schou M. (1981). Serum lithium concentrations around the clock with different treatment regimens and the diurnal variation of the renal lithium clearance. Acta Psychiatr. Scand. 64, 314–319. 10.1111/j.1600-0447.1981.tb00788.x 6801927

[B37] Lee J. H. Adler C. Norris M. Chu W. J. Fugate E. M. Strakowski S. M.. (2012). 4-T ^7^Li 3D MR spectroscopy imaging in the brains of bipolar disorder subjects. Magn. Reson. Med. 68, 363–368. 10.1002/mrm.24361 22692991 PMC3396736

[B38] Lukas R. J. Norman S. A. Lucero L. (1993). Characterization of nicotinic acetylcholine receptors expressed by cells of the SH-SY5Y human neuroblastoma clonal line. Mol. Cell. Neurosci. 4, 1–12. 10.1006/mcne.1993.1001 19912902

[B39] Luo J. Busillo J. M. Benovic J. L. (2008). M3 muscarinic acetylcholine receptor-mediated signaling is regulated by distinct mechanisms. Mol. Pharmcol. 74, 338–347. 10.1124/mol.107.044750 18388243 PMC7409535

[B40] Mathieu G. M. Denis S. Langelier B. Denis I. Lavialle M. Vancassel S. (2010). DHA enhances the noradrenaline release by SH-SY5Y cells. Neurochem. Int. 56, 94–100. 10.1016/j.neuint.2009.09.006 19770016

[B41] McCorvy J. D. Roth B. L. (2015). Structure and function of serotonin G protein-coupled receptors. Pharmacol. Ther. 150, 129–142. 10.1016/j.pharmthera.2015.01.009 25601315 PMC4414735

[B42] Mei L. Yamamura H. I. Roeske W. R. (1988). Muscarinic receptor-mediated hydrolysis of phosphatidylinositols in human neuroblastoma (SH-SY5Y) cells is sensitive to pertussis toxin. Brain Res. 447, 360–363. 10.1016/0006-8993(88)91140-7 3390705

[B43] Nakanishi S. Maeda N. Mikoshiba K. (1991). Immunohistochemical localization of an inositol 1,4,5-trisphosphate receptor, P400, in neural tissue: studies in developing and adult mouse brain. J. Neurosci. 11, 2075–2086. 10.1523/JNEUROSCI.11-07-02075.1991 1648604 PMC6575465

[B44] Nonaka S. Hough C. J. Chuang D.-M. (1998). Chronic lithium treatment robustly protects neurons in the central nervous system against excitotoxicity by inhibiting N-methyl-D-aspartate receptor-mediated calcium influx. Proc. Natl. Acad. Sci. U S A 95, 2642–2647. 10.1073/pnas.95.5.2642 9482940 PMC19446

[B45] Odagaki Y. Nishi N. Koyama T. (1997). Lack of interfering effects of lithium on receptor/G protein coupling in human platelet and rat brain membranes. Biol. Psychiatry 42, 697–703. 10.1016/s0006-3223(96)00443-x 9325563

[B46] Offermanns S. Simon M. I. (1995). Gα_15_ and Gα_16_ couple a wide variety of receptors to phospholipase C. J. Biol. Chem. 270, 15175–15180. 10.1074/jbc.270.25.15175 7797501

[B47] Offermanns S. Wieland T. Homann D. Sandmann J. Bombien E. Spicher K.. (1994). Transfected muscarinic acetylcholine receptors selectively couple to G_i_-type G proteins and G_q_/11. Mol. Pharmacol. 45, 890–898. 8190105

[B48] Peinado G. Osorno T. Gomez M. del. P. Nasi E. (2015). Calcium activates the light-dependent conductance in melanopsin-expressing photoreceptors of amphioxus. Proc. Natl. Acad. Sci. U S A 112, 7845–7850. 10.1073/pnas.1420265112 26056310 PMC4485138

[B50] Plenge P. Stensgaard A. Jensen H. V. Thomsen C. Mellerup E. T. Henriksen O. (1994). 24-hour lithium concentration in human brain studied by Li-7 magnetic resonance spectroscopy. Biol. Psychiatry 36, 511–516. 10.1016/0006-3223(94)90614-9 7827213

[B51] Renshaw P. F. Wicklund S. (1988). *In vivo* measurement of lithium in humans by nuclear magnetic resonance spectroscopy. Biol. Psychiatry 23, 465–475. 10.1016/0006-3223(88)90018-2 3125862

[B52] Ridley D. L. Pakkanen J. Wonnacott S. (2002). Effects of chronic drug treatments on increases in intracellular calcium mediated by nicotinic acetylcholine receptors in SH-SY5Y Cells. Br. J. Pharmacol. 135, 1051–1059. 10.1038/sj.bjp.0704508 11861334 PMC1573191

[B54] Sherman W. R. Leavitt A. L. Honchar M. P. Hallcher L. M. Phillips B. E. (1981). Evidence that lithium alters phosphoinositide metabolism: chronic administration elevates primarily D-myo-inositol-l-phosphate in cerebral cortex of the rat. J. Neurochem. 36, 1947–1951. 10.1111/j.1471-4159.1981.tb10819.x 6264039

[B55] Smith F. E. Thelwall P. E. Necus J. Flowers C. J. Blamire A. M. Cousins D. A. (2018). 3D ^7^Li magnetic resonance imaging of brain lithium distribution in bipolar disorder. Mol. Psychiatry 23, 2184–2191. 10.1038/s41380-018-0016-6 29426954 PMC5955212

[B56] Soares J. C. Boada F. Keshavan M. S. (2000). Brain lithium measurements with (7) Li magnetic resonance spectroscopy (MRS): a literature review. Eur. Neuropsychopharmacol. 10, 151–158. 10.1016/s0924-977x(00)00057-2 10793316

[B57] Sonnier H. Kolomytkina O. Marino A. (2003). Action potentials from human neuroblastoma cells in magnetic fields. Neurosci. Lett. 337, 163–166. 10.1016/s0304-3940(02)01329-0 12536049

[B58] Sourial-Bassillious N. Rydelius P. A. Aperia A. Aizman O. (2009). Glutamate mediated calcium signaling: a potential target for lithium action. Neuroscience 161, 1126–1134. 10.1016/j.neuroscience.2009.04.013 19362133

[B59] Sousa S. R. Vetter I. Ragnarsson L. Lewis R. J. (2013). Expression and pharmacology of endogenous Cav channels in SH-SY5Y human neuroblastoma cells. PLoS One 8:e59293. 10.1371/journal.pone.0059293 23536870 PMC3607609

[B60] Timmer R. T. Sands J. M. (1999). Lithium intoxication. J. Am. Soc. Nephrol. 10, 666–674. 10.1681/ASN.V103666 10073618

[B61] Toselli M. Masetto S. Rossi P. Taglietti V. (1991). Characterization of a voltage-dependent calcium current in the human neuroblastoma cell line SH-SY5Y during differentiation. Eur. J. Neurosci. 3, 514–522. 10.1111/j.1460-9568.1991.tb00838.x 12106483

[B62] Toselli M. Tosetti P. Taglietti V. (1996). Functional changes in sodium conductances in the human neuroblastoma cell line SH-SY5Y during *in vitro* differentiation. J. Neurophysiol. 76, 3920–3927. 10.1152/jn.1996.76.6.3920 8985889

[B63] Tosetti P. Taglietti V. Toselli M. (1998). Functional changes in potassium conductances of the human neuroblastoma cell line SH-SY5Y during *in vitro* differentiation. J. Neurophysiol. 79, 648–658. 10.1152/jn.1998.79.2.648 9463428

[B64] Van Acker K. Nadif Kasri N. De Smet P. Parys J. B. De Smedt H. Missiaen L.. (2002). IP_3_-mediated Ca^2+^ signals in human neuroblastoma SH-SY5Y cells with exogenous overexpression of type 3 IP_3_ receptor. Cell Calcium 32, 71–81. 10.1016/s0143-4160(02)00092-1 12161107

[B65] Vetter I. Mozar C. A. Durek T. Wingerd J. S. Alewood P. F. Christie M. J.. (2012). Characterisation of Na(v) types endogenously expressed in human SH-SY5Y neuroblastoma cells. Biochem. Pharmacol. 83, 1562–1571. 10.1016/j.bcp.2012.02.022 22410003

[B66] Wang H. Y. Friedman E. (1999). Effects of lithium on receptor-mediated activation of G proteins in rat brain cortical membranes. Neuropharmacology 38, 403–414. 10.1016/s0028-3908(98)00197-x 10219978

[B67] Watanabe M. Nakamura M. Sato K. Kano M. Simon M. I. Inoue Y. (1998). Patterns of expression for the mRNA corresponding to the four isoforms of phospholipase Cβ in mouse brain. Eur. J. Neurosci. 10, 2016–2025. 10.1046/j.1460-9568.1998.00213.x 9753089

[B68] Wu D. Katz A. Lee C. H. Simon M. I. (1992). Activation of phospholipase C by α_1_-adrenergic receptors is mediated by the α subunits of Gq family. J. Biol. Chem. 267, 25798–25802. 10.1016/S0021-9258(18)35680-1 1334487

